# A surface metal ion-modified 3D-printed Ti-6Al-4V implant with direct and immunoregulatory antibacterial and osteogenic activity

**DOI:** 10.3389/fbioe.2023.1142264

**Published:** 2023-03-16

**Authors:** Yipeng Wu, Xiangwen Shi, Jianjun Wang, Yang Li, Jiang Wu, Daqi Jia, Yan Bai, Xiaopei Wu, Yongqing Xu

**Affiliations:** ^1^ Graduate School, Kunming Medical University, Kunming, China; ^2^ Laboratory of Yunnan Traumatology and Orthopedics Clinical Medical Center, Yunnan Orthopedics and Sports Rehabilitation Clinical Medicine Research Center, Department of Orthopedic Surgery, 920th Hospital of Joint Logistics Support Force, Kunming, China; ^3^ State Key Laboratory of Advanced Technology for Materials Synthesis and Processing, Wuhan University of Technology, Wuhan, China

**Keywords:** metal ion, 3D-printed Ti-6Al-4V, immunoregulatory antibacterial activity, immunoregulatory osteogenic activity, bone infection

## Abstract

The high concentration of antibacterial metal ions may exhibit unavoidable toxicity to cells and normal tissues. The application of antibacterial metal ions to activate the immune response and induce macrophages to attack and phagocytose bacteria is a new antimicrobial strategy. Herein, 3D-printed Ti-6Al-4V implants modified by copper, and strontium ions combined with natural polymers were designed to treat implant-related infections and osseointegration disorders. The polymer-modified scaffolds rapidly released a large amount of copper and strontium ions. During the release process, copper ions were employed to promote the polarization of M1 macrophages, thus inducing a proinflammatory immune response to inhibit infection and achieve the immune antibacterial activity. Meanwhile, copper and strontium ions promoted the secretion of bone-promoting factors by macrophages, induced osteogenesis and showed immunomodulatory osteogenesis. This study proposed immunomodulatory strategies based on the immunological characteristics of target diseases and provided ideas for the design and synthesis of new immunoregulatory biomaterials.

## Introduction

Segmental bone defects caused by high-energy trauma, infection, bone tumors and congenital malformations still pose great challenges to orthopedic surgeons. The most common treatment includes autogenous and allogeneic bone transplantation, both of which have their own disadvantages. Autogenous bone transplantation requires additional surgical operations, which leads to more trauma and postoperative complications, such as nerve injury, fracture and infection in the bone donor area. Allogeneic bone transplantation has the risk of immune disease and slow bone remodeling (Dou et al., 2016; Li et al., 2016; Wu et al., 2018). 3D-printed porous titanium, a potential bone substitute material, possesses good mechanical properties, appropriate elastic modulus and excellent corrosion resistance. The porous structure simulates natural bone, which is beneficial to the formation of new bone and provides a new method for the treatment of bone defects (Puleo and Nanci, 1999; Esen et al., 2016; Ma et al., 2018; Tsai et al., 2019; Walsh et al., 2019).

The high porosity and connectivity of porous bone reconstruction implants and corresponding personalized customization can be manufactured by 3D printing technology (Feng et al., 2021). However, the printed scaffold without surface modification may exhibit poor adhesion ability owing to insufficient cell adhesion sites. 3D-printed titanium scaffolds comprise good biocompatibility, but their deficient bone inductivity reduces the bone binding properties or stability of composite structural organization (Wang et al., 2022). In severe infection cases, the implantation may suffer from loosening or even fall off. In addition, the lack of antibacterial ability on the scaffolds may induce a high incidence of implant infection and peripheral inflammation (Jia et al., 2019). Therefore, it is necessary to enhance the antimicrobial properties of the implant surface while improving the osseointegration ability of the titanium alloy bone tissue engineering scaffold.

Currently, the surface modification methods of biomaterials to achieve antimicrobial and osteogenic activity include sandblasting acid etching, ultrasonic microarc oxidation, plasma spraying, and laser treatment (Bütev et al., 2016; Choi and Park, 2018). For the implantation with porous structure and complex sharp, inappropriate modification may lead to uneven surface coating, the formation of sharp edges and corners, dissolution of titanium ions, and other defects (Kwon et al., 2006; Shishkovsky and Morozov, 2013). The coating materials are prepared into a sol and uniform coating and an anhydrous gel surface is formed on the metal surface through solvent evaporation and agglomeration, which is considered to be an effective modification method. Under alkaline conditions, the catecholamine functional group and lysine terminal amino group of dopamine form a strong adhesive dopamine (PDA) film on the surface of the glass, metal, ceramic, organic materials, etc. After self-polymerization, dopamine can chelate Ag ions on the surface of adhesive materials and provide a stable antibacterial coating by releasing silver ions (Saidin et al., 2013; Zhang et al., 2018). Alginate has the characteristics of non-toxicity, high absorption, gel formation and oxygen permeability, as well as promoting wound healing and high ion adsorption with good biocompatibility (Rastogi and Kandasubramanian, 2019; He et al., 2022). After the self-assembly of sodium alginate and dopamine by electrostatic interactions, the polyelectrolyte complex is formed by the combined action of metal ions (copper and strontium), which method can be used to modify the surface of titanium alloys to realize antibacterial and bone-promoting functions.

Additionally, drugs or materials are employed to activate the immune response and induce macrophages to attack and phagocytose bacteria, thus achieving the antimicrobial purposes (Seebach and Kubatzky, 2019; Dong et al., 2022). Previous studies reported that copper is closely related to human immune regulation and may affect the immune system. Huang et al. (Huang et al., 2019) demonstrated that copper ions enhanced bactericidal and bacterial phagocytosis by activating macrophages when loaded on the surface of the biomaterials. As an essential osteophilic trace element for the human body, strontium ions induce and maintain the anti-inflammatory microenvironment, which is conducive to inhibiting the inflammatory response and promoting tissue repair (Cai et al., 2021; You et al., 2022). Therefore, copper and strontium ions can be introduced into the implant materials to regulate the immune (Barbeck et al., 2022).

**SCHEME 1:**
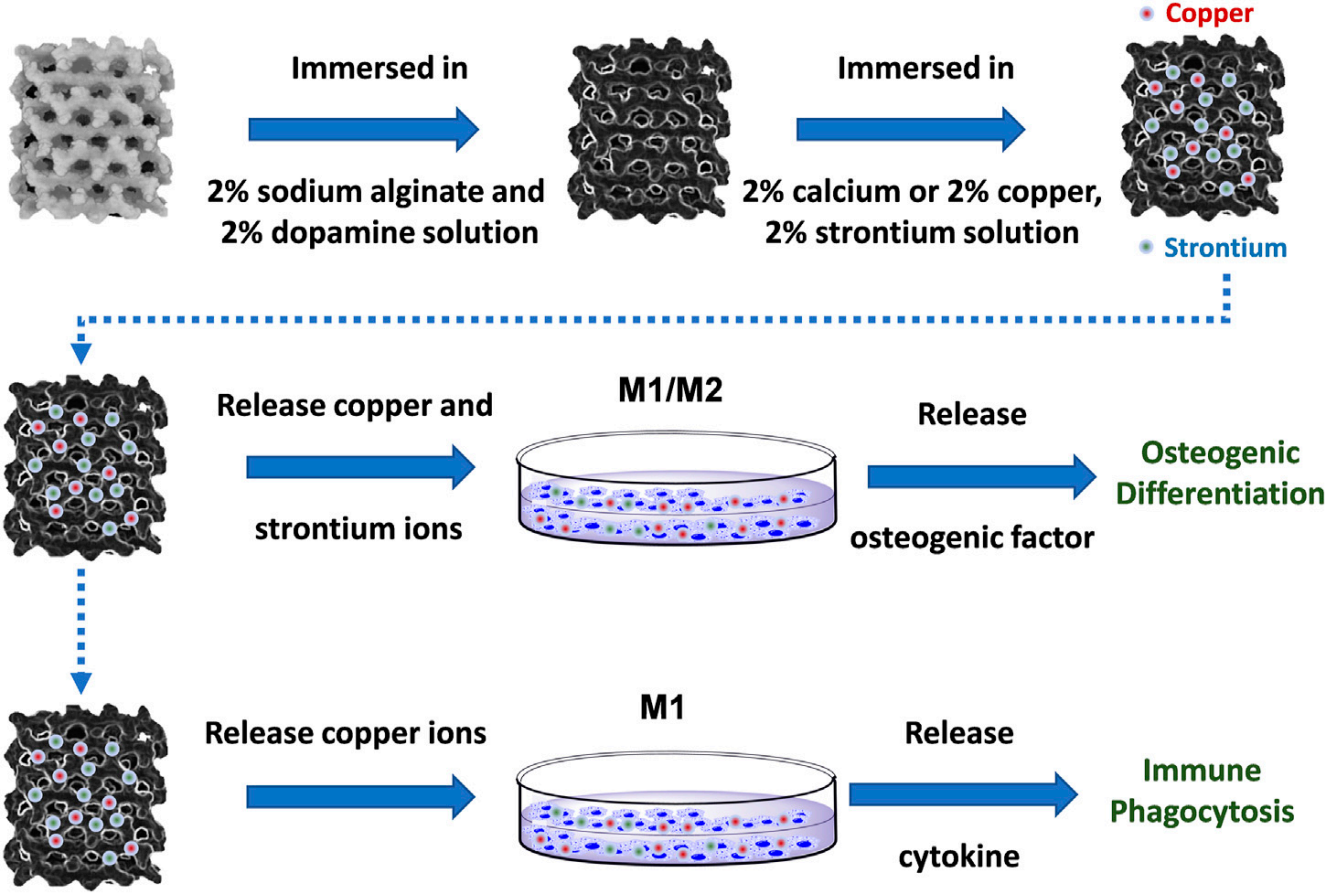
The fabrication and evaluation of metal ion-modified Ti-6Al-4V implants.

In this study, 3D-printed Ti-6Al-4V implants modified by copper, and strontium ions combined with natural polymers were designed to treat implant-related infections and eliminate osseointegration disorders. The polymer-modified scaffolds rapidly released copper and strontium ions in large quantities, which targeted macrophages by mediating immune responses to attack and phagocytose bacteria. During the process, copper ions were used to promote the polarization of M1 macrophages, induce a proinflammatory immune response to inhibit infection and achieve immune antibacterial activity. Meanwhile, copper and strontium ions both promoted macrophages to the secretion of bone-promoting factors and induced immunomodulatory osteogenesis (Scheme 1). The design and synthesis of Cu-Sr modified 3D printed scaffolds provide a novel strategy for immunomodulation and treatment of bone infections.

## Materials and methods

### 3D printing of Ti-6Al-4V implants

The scaffolds with a cylinder shape (φ3.0 × 3.0 cm) were designed using EOSTATE Magics RP software (Materialise), and the unit of the internal pore was a diamond (80% porosity). The digital files of the four scaffolds were saved as the Standard Tessellation Language (STL) file format. The STL files were imported into an SLM 3D printer (EOSINT M 280, P. R. China). The power of the printer was 200 W, the scanning pitch was 0.08 mm, the scanning speed was 7 m/s, and the thickness of the powder layer was 30 μm.

### Fabrication of metal ion-modified Ti-6Al-4V implants

3D-printed of Ti-6Al-4V implants were immersed in 2% sodium alginate and 2% dopamine solution and stirred for 4 h. The 3D printing of Ti-6Al-4V implants was removed, and 2% calcium, copper, and strontium solution was prepared. The solution was evenly coated on the 3D-printed Ti-6Al-4 V implant surface with the precrosslinking. Then, the precrosslinked implants were immersed in the above solution for 4 h, and the support was removed and dried.

### Characterization of metal ion-modified Ti-6Al-4V implants

The surface chemical composition of the metal ion-modified Ti-6Al-4V implants was analyzed by a Fourier transform infrared spectroscopy (Thermo Fisher IN10, USA) in the 400-4,000 cm^-1^ range (Supplementary Figure S1). The surface topography analysis of the metal ion-modified Ti-6Al-4V implants was achieved using a scanning electron microscope (TESCAN MIRA LMS, Czech Republic). The surface element mapping of the metal ion-modified Ti-6Al-4V implants was observed using an EDS energy spectrometer with a scanning electron microscope (TESCAN MIRA LMS, Czech Republic). Skyscan 1176 micro-CT (Bruker, Kontich, Belgium) was performed to collect data from samples at the Institute of Hydrobiology, Chinese Academy of Sciences (Wuhan, China). The compressive strength test was mainly based on a porous model (φ3 × 3). The samples were placed on the pressure table of a universal material testing machine (CMT6103, USA), and the loading speed of the indenter was set to 1 mm/min. The degradation of metal ion-modified Ti-6Al-4V implants was investigated *in vitro* by immersing the samples in phosphate-buffered saline (PBS), the content of metal ions was measured by inductively coupled plasma atomic emission spectrometry (Agilent 720ES, USA).

### Cell culture

The murine preosteoblast cell line (MC3T3-E1) was purchased from the BeNa Culture Collection (Henan, China). MC3T3-E1 cells were seeded in α-MEM (Gibco) supplemented containing 10% fetal bovine serum (Gibco) and 1% penicillin and streptomycin (Gibco), and the medium was replaced every 3 days. To induce osteogenic differentiation, a confluent monolayer of MC3T3-E1 cells was treated with 10 nM dexamethasone (Sigma) and 0.2 mM ascorbic acid (Sigma), with a medium change every 3 days. An equivalent amount of vehicle was used as a control when needed. Mouse mononuclear macrophage cells (RAW264.7) were purchased from Tongji Medical College Huazhong University of Science and Technology (Wuhan, China). RAW264.7 cells were cocultured with metal ion-modified Ti-6Al-4V implants to prepare a conditioned medium. The complete medium used for RAW264.7 cells includesd 500 ml high glucose medium (Gibco), 10 ml fetal bovine serum (Gibco), and 5 ml of penicillin/streptomycin solution (Gibco). The medium was changed every 2 days during cell culture.

### Cytocompatibility and immune response

The viability of RAW264.7 cells was assessed by staining with Calcein-AM according to the manufacturer’s instructions. For cell morphology and spreading observation, RAW264.7 cells were identified by TRITC phalloidin and DAPI for cytoskeleton and cellular nuclei staining. RAW264.7 cells were observed using an inverted fluorescence microscope (Olympus, Olympus IX71, Japan). RNA was extracted according to the instructions of the RNA extraction kit. The extracted RNA was then reverse transcribed into cDNA, and gene expression was detected by a Light Cycler PCR machine (Roche) using Fast Start Universal SYBR Green Master Mix (Roche). The primer sequences were as follows: MIF (forward: 5′-CTT​TGT​ACC​GTC​CTC​CGG​TC-3′, reverse: 5′- CGT​TCG​TGC​CGC​TAA​AAG​TC-3′), NF-κB (forward: 5′- GGA​GGC​ATG​TTA​GTG​G-3′, reverse: 5′-CCCTG CGTTGGATTTCGTG-3′), IKK (forward: 5′-GTG​TGT​GCT​AAC​CGT​TAC​CT-3′, reverse: 5′- GCT​CTT​AGC​ACA​GAC​ATT​GGA​AG-3′), CD86 (forward: 5′-AGC​ACG​GAC​TTG​AAC​AAC​CA-3′, reverse: 5′- TGT​AAA​TGG​GCA​CGG​CAG​AT-3′), OSM (forward: 5′-CAA​TCG​TGG​CTG​CTC​CAA​CTC​T-3′, reverse: 5′- GGT​GTG​TTC​AGG​TTT​TGG​AGG​C-3′), BMP-2 (forward: 5′- TTT​TGG​TCA​CGA​TGG​GAA​GGG-3′, reverse: 5′-ACA​ATC​CAG​TCG​TTC​CAC​CC-3′), and GAPDH (forward: 5′-GCA​GTG​GCA​AAG​TGG​AGA​TT-3′, reverse: 5′- TCT​CCA​TGG​TGG​TGA​AGA​CA-3′), Immune osteogenesis *in vitro*.

MC3T3-E1 cells were seeded on 6-well plates. When the cells were 80% confluent, the medium was replaced with conditioned medium and osteogenic differentiation medium (1:1) after 2 days. The cells cultured for 7 days were fixed with 4% paraformaldehyde for 10 min, stained with an Alkaline Phosphatase Assay Kit (Colorimetric, ab83369) at 37°C for 1 h in the discarded fixative, and finally photographed under a microscope. The cells cultured for 14 days were lysed in RIPA buffer. The protein concentrations in the cell lysates were determined by using a protein quantitative detection assay kit. The total proteins in cell lysates were separated by SDS‒PAGE. After electrophoresis, the protein bands were transferred to a PVDF membrane by using a liquid transblot system (Bio-Rad). The 0.45 µm membranes used for the immunodetection of proteins were blocked with 5% skim milk. Diluted primary antibodies (OPN, RUNX2, Collagen I, Smad1, Smad5, Smad8, 1:800) bound to the membrane were detected with secondary antibodies diluted 1:2000. Data were collected by ECL color rendering. The cells cultured for 21 days were fixed with 4% paraformaldehyde for 10 min, stained with 1% alizarin red at 37 °C for 1 h after discarding the fixed solution, and finally photographed under a microscope.

### Immune antibacterial *in vitro*

RAW264.7 macrophages were cultured in 6-well plates with scaffolds (+RAW-CM), and the control group received only an equivalent amount of culture medium without macrophages (-RAW-CM) for 48 h at 37°C. Then, calcein-AM labeled *S. aureus* was added to the 6-well plate. After incubation for 24 h, the phagocytosis of RAW264.7 cells against *S. aureus* was observed by an inverted fluorescence microscope (Olympus, Olympus IX71, Japan). The treated bacterial suspension was used to observe the number of *S. aureus* not swallowed by an inverted fluorescence microscope (Olympus, Olympus IX71, Japan). The sample was diluted 10^6^ times with PBS and 200 μl was taken and dropped onto the LB solid medium plate and smeared evenly with a triangular coating rod. The cells were incubated in a constant temperature incubator at 37°C for 24 h and then counted.

### Statistical analysis

At least three independent samples in each group of experimental data were analyzed with statistical software SPSS18.0, with a mean ± standard deviation (n = 3), and groups were compared through one-way analysis of variance (ANOVA) to determine the significant difference between test groups. The statistical difference was marked with an asterisk (*), and set the statistical difference was set at *p* < 0.05.

## Results

### Characteristics of metal ion-modified Ti-6Al-4V implants

**FIGURE 1:**
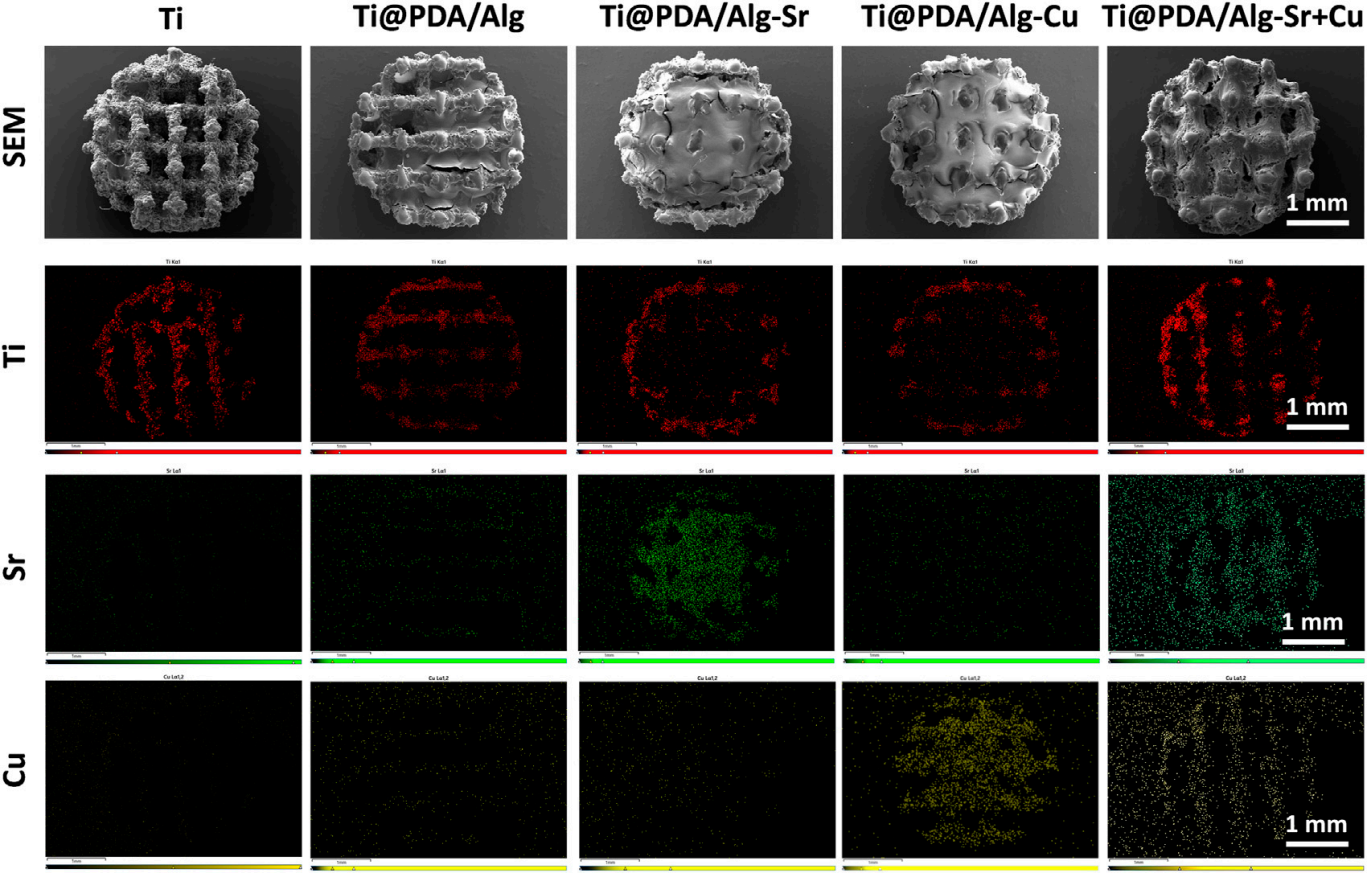
SEM image and EDX elemental mapping images of the metal ion-modified Ti-6Al-4V implants.

The changes in the sample surface after surface modification were observed by SEM, as shown in Figure 1. The 3D-printed titanium alloy showed a uniform porous structure with a burr-like appearance. The pore space of the surface was filled after the polymer build-up process, indicating that the dopamine and sodium alginate-based layers were successfully coated. EDS test was performed to illustrate the chemical composition of the surface coating. As shown in the surface scan of Figure 1, color signals appeared for different groups of copper and strontium, which proved that copper and strontium were modified onto the titanium surface by polymers.

**FIGURE 2:**
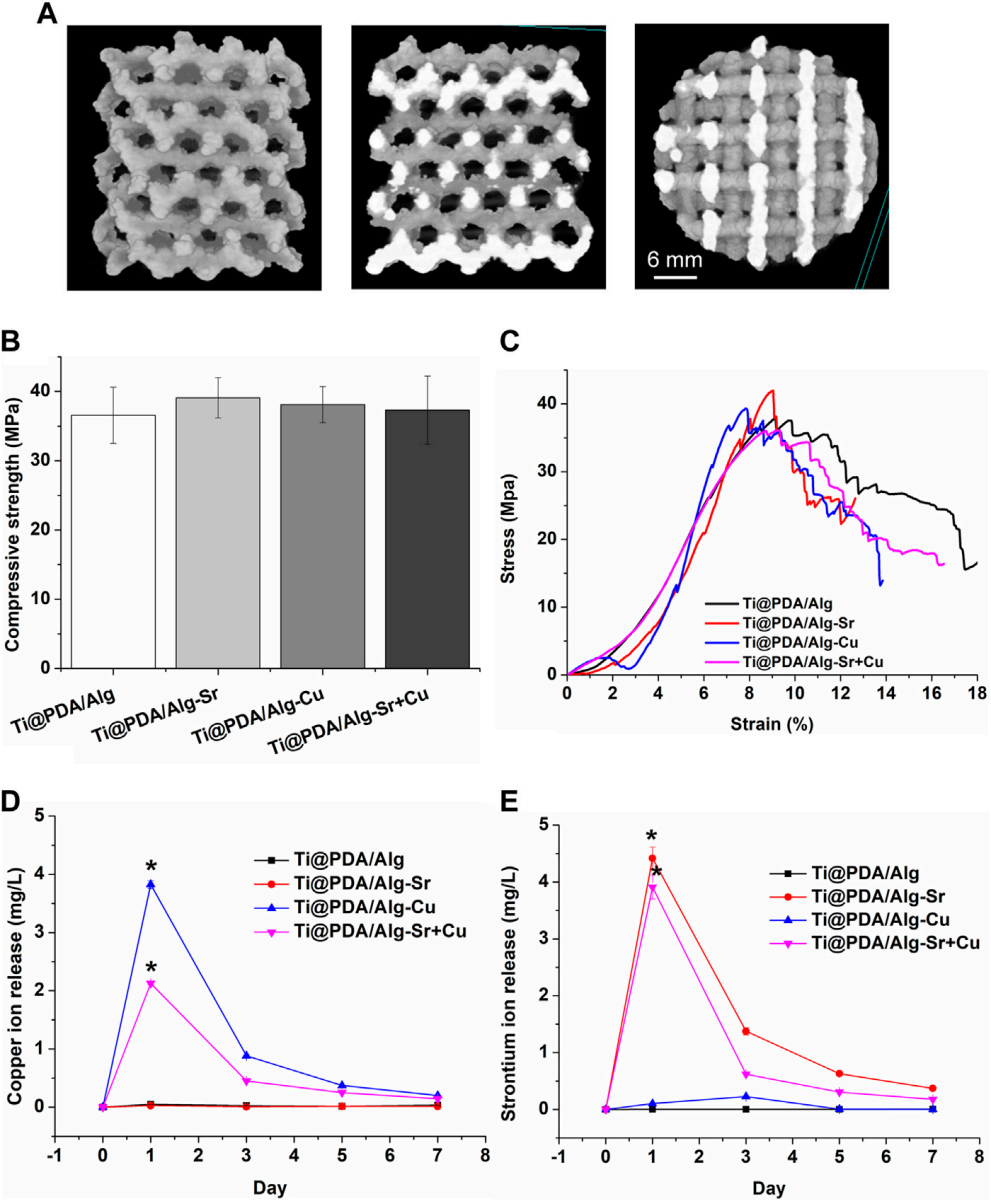
Characterization of the metal ion-modified Ti-6Al-4V implants. **(A)** Micro CT; **(B)** Compressive strength; **(C)** Stress-strain curve; **(D)** Copper ion release curve; **(E)** Strontium ion release curve.

The micro-CT in Figure 2A shows that the diamond-based models with good mechanical properties were employed in the porous structure and served as a component unit of the scaffold, enabling the scaffold to have good mechanical strength. The solid model is a cylinder with a diameter of 3 mm, a height of 5 mm, and a pore diameter of 500 μm. The compression performance of the macroscopic porous scaffold with a random pore structure prepared on the surface is in Figures 2B, C. At the initial stage of loading, the elastic modulus of each porous support was small. At the stage of elastic deformation, some samples exhibited jagged changes, which was caused by a small number of printing defects in the porous scaffold and mechanical weak points, The compressive strength of the scaffold was not affected by the surface modification. The release curve of ions (Figures 2D, E) showed that the explosive release of copper and strontium ions from the coating occurred in the first 3 days. The release rate slowed down significantly and remained stable in the next 3 days until it was completed within a week.

### Influences of metal ion-modified Ti-6Al-4V implants on macrophage behavior

**FIGURE 3:**
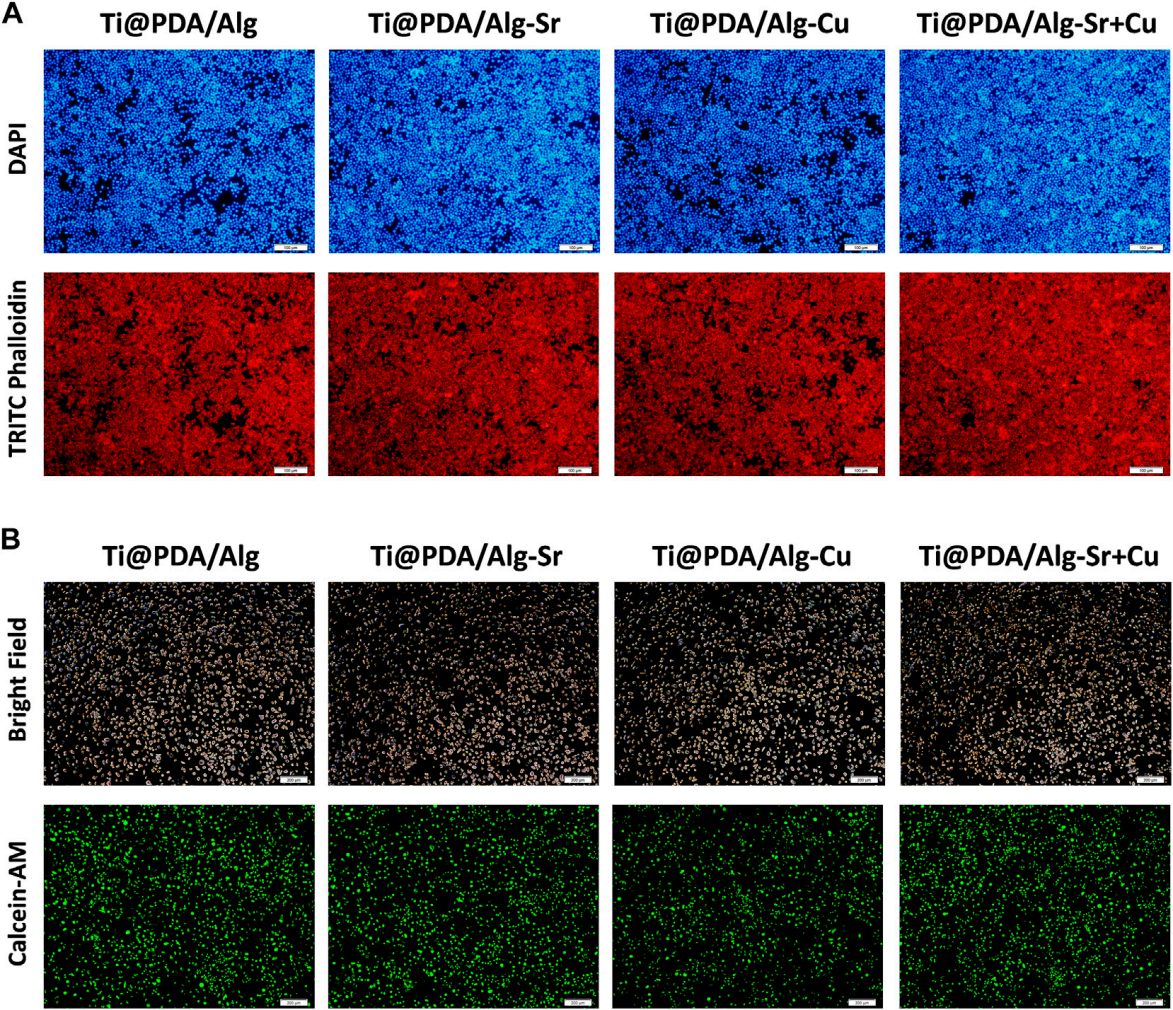
Cytocompatibility of the metal ion-modified Ti-6Al-4V implants. **(A)** Nuclear (Blue) membrane (Red) staining, scale bar = 100 μm; **(B)** Cell viability staining (Green), scale bar = 200 μm.

After coculturing RAW264.7 cells with scaffolds for 2 days, Figure 3A demonstrated that the actin fluorescence stained with rhodamine-labeled phalloidin was red. The shape of the nucleus stained by 4-diamino-6-diamino-2-phenylindole (DAPI) was complete and clear, indicating that the cells were in excellent condition, with overlapping growth and mutual adhesion. Furthermore, the groups of cocultured cells were labeled with calcein, and the fluorescence inside the cells was stronger, suggesting good metabolic activity (Figure 3A).

**FIGURE 4:**
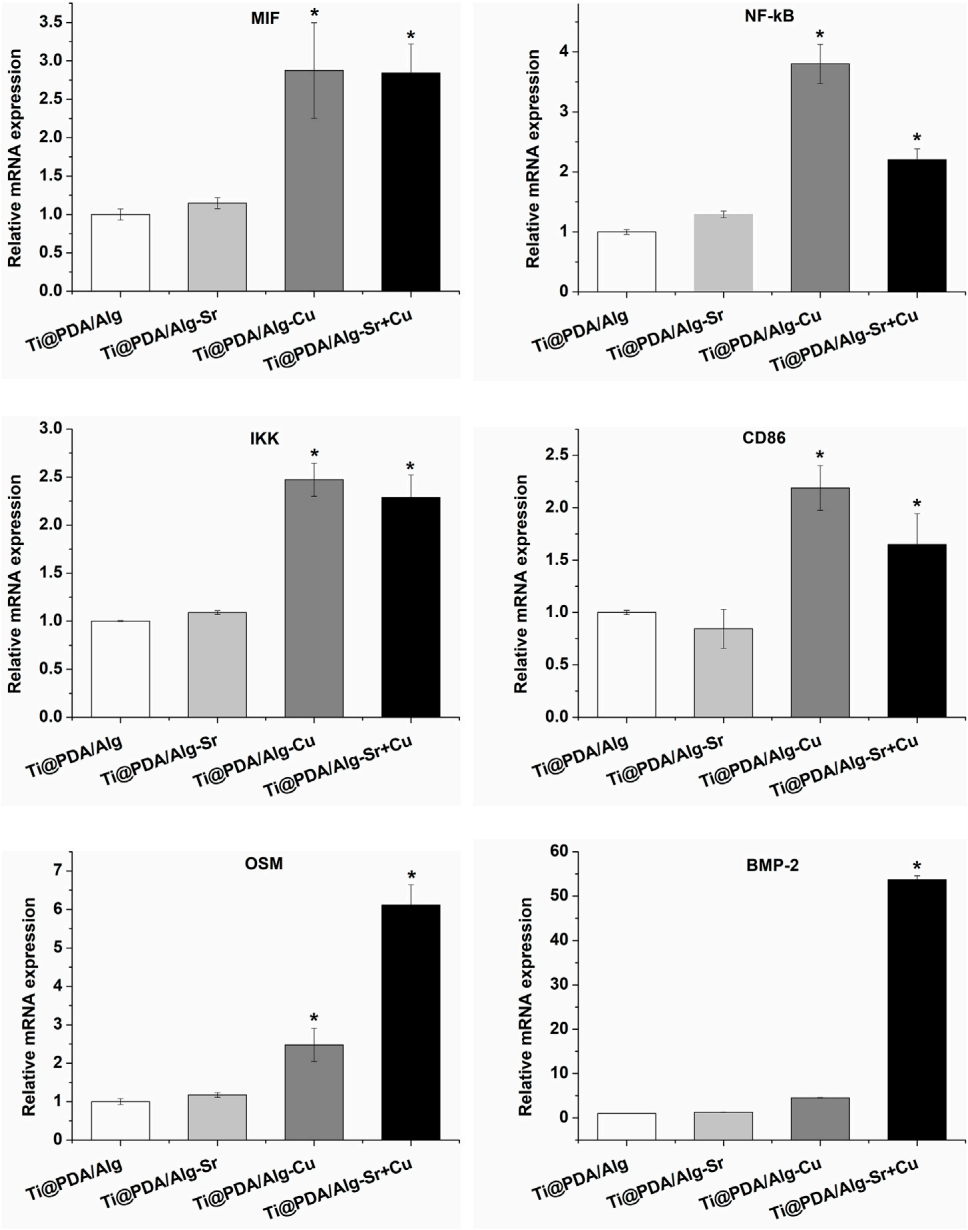
Immune reaction of the metal ion-modified Ti-6Al-4V implants.

Additionally, the levels of inflammation-associated cytokine mRNA after the coculture of RAW264.7 cells and scaffolds were measured by RT-PCR. As shown in Figure 4, the expression level of proinflammatory cytokines (such as MIF, NF-kB, and IKK) in the Cu and Cu-Sr groups were significantly upregulated at 2 days. In contrast, there was no significant change in proinflammatory cytokines in the Sr group at 2 days. The cell phenotypic marker CD86 in both the Cu and Cu-Sr groups remained high at 2 days. However, the expression levels of CD86 in the Sr group did not change significantly at 2 days. Bone morphogenetic protein-2 (BMP-2) and tumor suppressor M (OSM) are effective inducers of osteogenesis secreted by macrophages. The expression of BMP-2 and OSM factors in the Cu-Sr group was high at 2 days, while it did not change significantly in the Cu and Sr groups. Overall, the Cu and Cu-Sr groups were equipped with proinflammatory and bone-enhancing effects.

### Immune osteogenic effect of metal ion-modified Ti-6Al-4V implants

**FIGURE 5:**
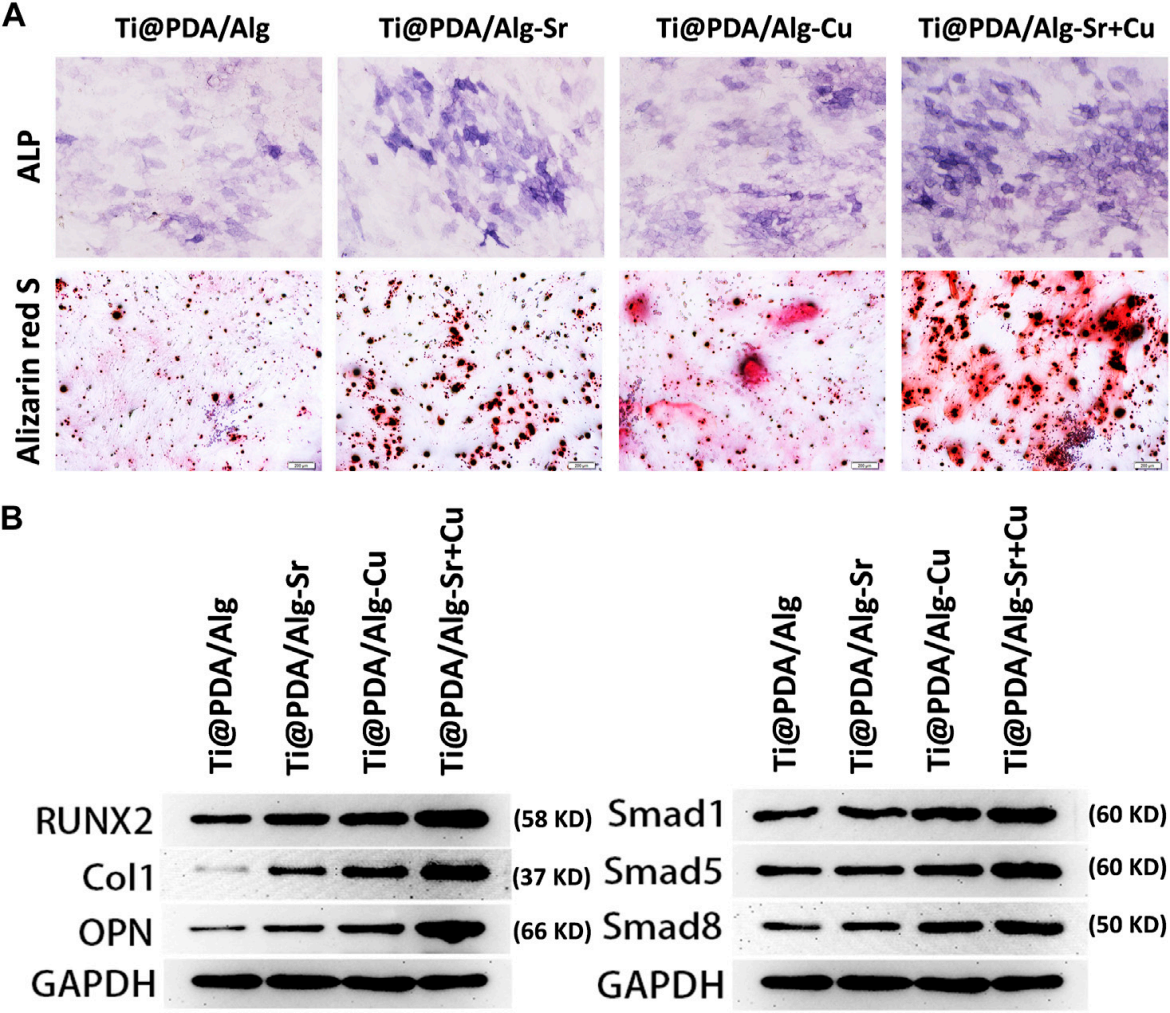
Immune osteogenic effect of the metal ion-modified Ti-6Al-4V implants. **(A)** Alkaline phosphatase staining and alizarin red s staining, scale bar = 200 μm; **(B)** Osteogenic differentiation marker protein and BMP signal pathway.

MC3T3-E1 cells were seeded on different scaffolds and incubated for 24 h, and conditioned cultures were prepared. The osteogenic differentiation of the MC3T3-E1 cells was evaluated after incubating with scaffolds for 7 days. Alkaline phosphatase (ALP) is considered an early marker of osteogenic differentiation, and the ALP expression in MC3T3 cells was first assessed by ALP immunofluorescence staining. Figure 5A indicates a significant increase in ALP staining in the Cu-Sr group at 7 days. Consistent with the ALP staining results, the Cu-Sr group possessed more calcified nodules than other groups (Figure 5A). The expression levels of osteogenic genes (RUNX2, COL-1, OPN) were determined by Western blotting. Compared with the control group, the proteins in the Sr, Cu and Cu-Sr groups were all upregulated to some extent, in which the protein expression in the Cu-Sr group was significantly increased (Figure 5B). Overall, the Cu-Sr modified 3D-printed scaffold exhibited the ability to promote osteogenic differentiation by modulating macrophages. Compared with the control group, the identification of BMP-2 Smad1/5/8 signaling pathway showed that the proteins in the Sr, Cu and Cu-Sr groups were all upregulated to some extent, and the Cu-Sr group was more prominent (Figure 5B), suggesting that the Cu-Sr modified 3D-printed scaffold regulated immune osteogenesis through the BMP-2 signaling pathway.

### Immune antibacterial effect of metal ion-modified Ti-6Al-4V implants

**FIGURE 6:**
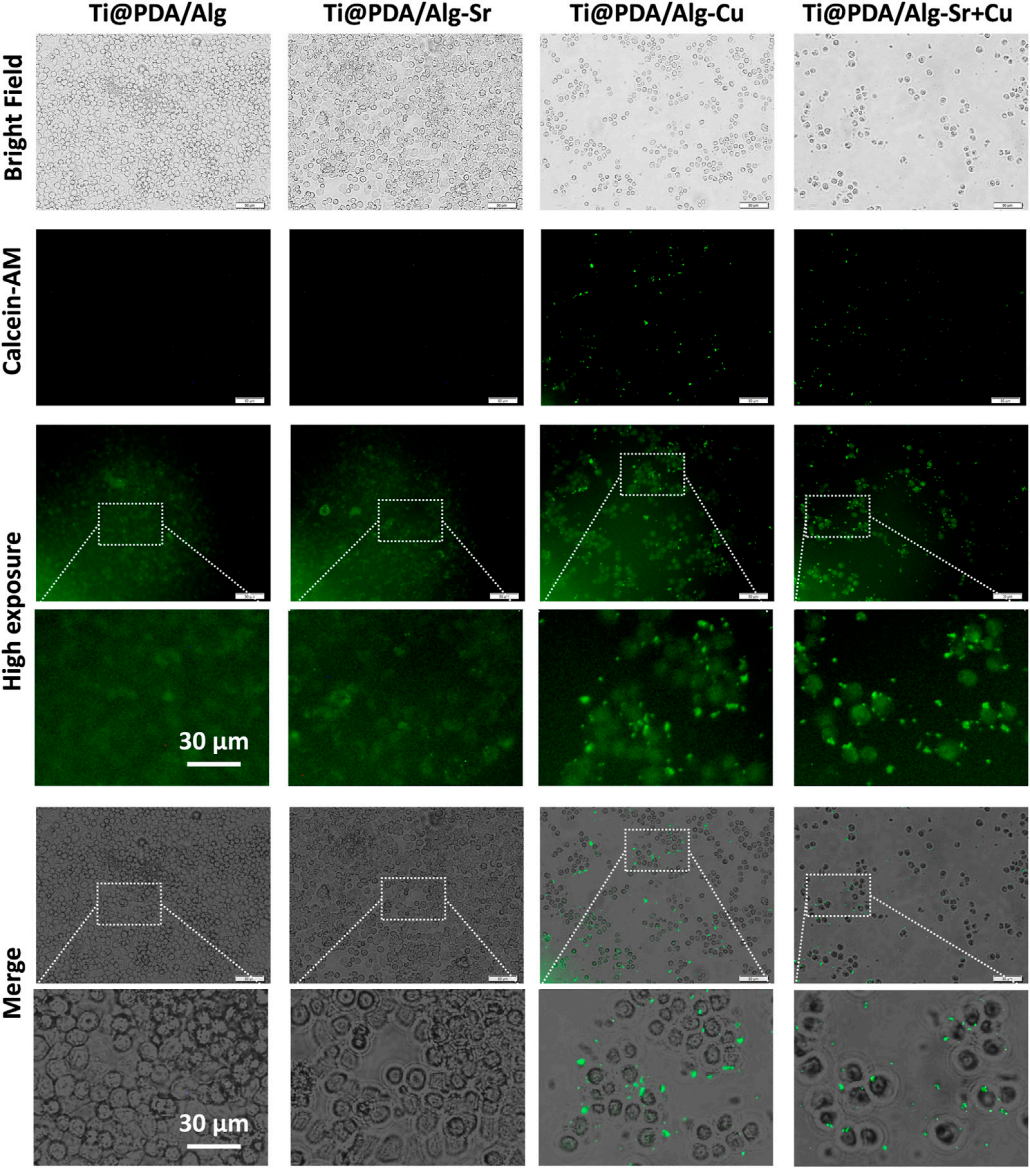
Immune phagocytosis of the metal ion-modified Ti-6Al-4V implants. Scale bar = 50 μm.

**FIGURE 7:**
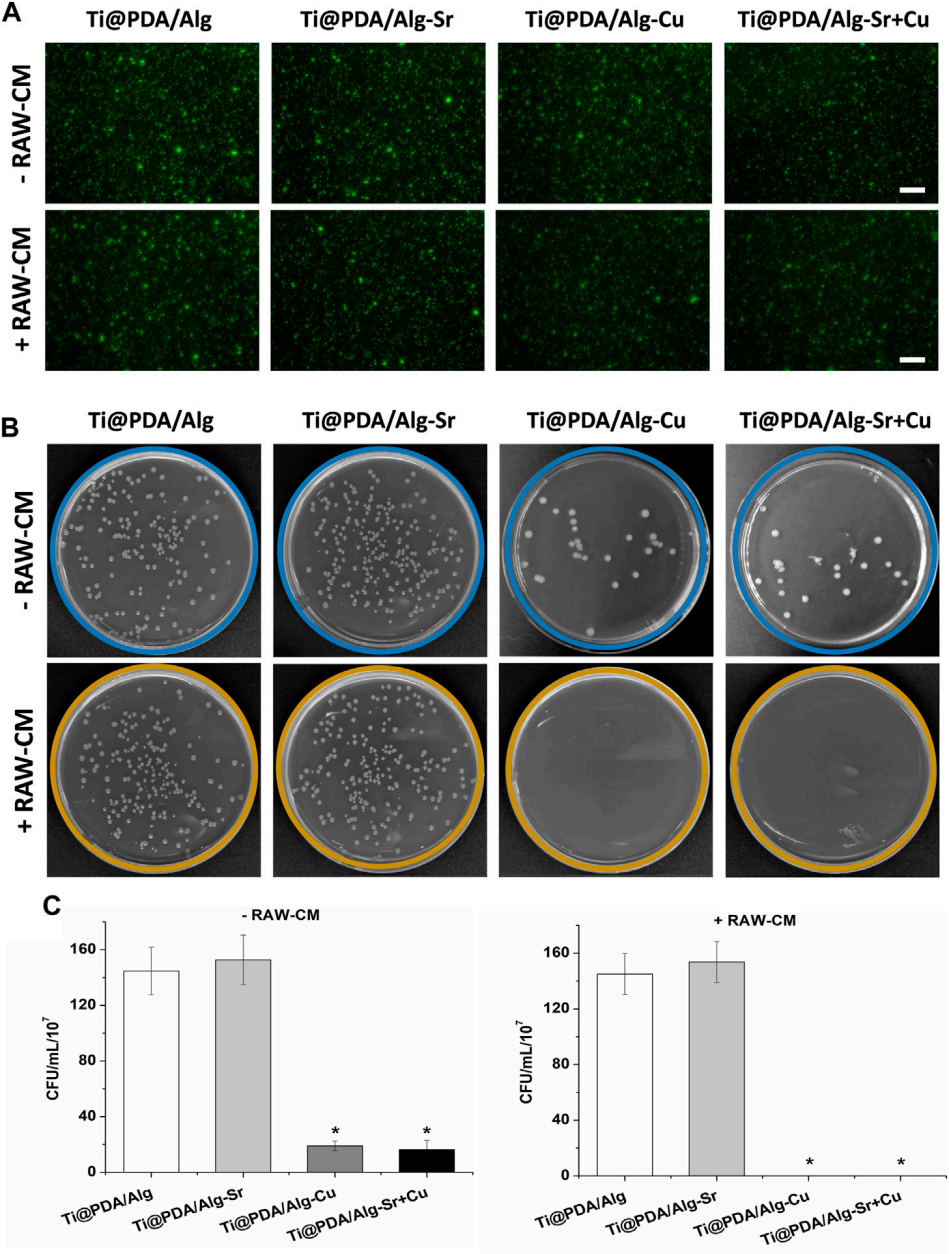
Count of bacteria not swallowed. **(A)** Bacterial activity staining (Green), Scale bar = 50 μm; **(B)** Bacterial coating plate growth; **(C)** Bacterial coating count. +RAW-CM: Supplemented with RAW macrophage culture medium, -RAW-CM: Not supplemented with RAW macrophage culture medium.

The effect of the Cu-Sr modified 3D-printed scaffold on the antibacterial properties after regulating the M1 polarization of macrophages was investigated. As shown in Figure 6, RAW264.7 macrophages were seeded on Cu and Cu-Sr-modified 3D-printed scaffolds, incubated for 2 days, and cocultured with bacteria for 1 day. In the Cu and Cu-Zn groups, the phagocytic activity of macrophages was significantly enhanced at 2 days. More *S. aureus* could be captured and swallowed based on the number of fluorescence labeled cells. Further detection by calcein staining and plate counts showed that fewer *S. aureus* was isolated from the coculture system, indicating that macrophages treated with Cu and Cu-Sr modified 3D-printed scaffolds captured and phagocytosed more *S. aureus* (Figures 7A–C).

## Discussion

Implant-associated infection and implant osseointegration disorders are catastrophic complications after total joint replacement (Kong et al., 2017). Once infection occurs in the prosthesis, the bacteria escape from the immune attack or develop immune tolerance when the intensity of the inflammatory response is insufficient. The above characteristics of bacteria lead to severe infection (Kavanagh et al., 2018). Under infected conditions, a functional imbalance between osteoblasts and osteoclasts leads to bone remodeling and bone loss, which may further result in prosthesis loosening or loss (Nasser et al., 2022). Therefore, simultaneous antimicrobial and pro-bone repair should be emphasized to cope with implant-associated infections. In this study, a 3D-printed scaffold modified by Cu-Sr was proposed and designed, which provides a new idea for bone infection repair from the perspective of activating the immune response while promoting its antibacterial and osteogenic abilities.

Copper ions play an important role in the innate immune system, which induces macrophage polarization to the M1 phenotype and enhances the secretion of proinflammatory cytokines (IL-6 and TNF-α) (Shi et al., 2016). Previous studies have proven that supplementation with copper ions enhanced the phagocytosis and the intracellular bactericidal ability of macrophages (White et al., 2009; Festa et al., 2014). Mechanistically, the addition of copper ions to the culture medium or release from the surface of biomaterials can polarize macrophages to a pro-inflammatory and pro-fibrotic M1 phenotype by activating copper transporter protein 1 (CTR1) and Na,K-ATPases (ATP1A) in macrophages, enhancing the ability of macrophages to capture and phagocytize bacteria (Huang et al., 2019). The polarization of M1 macrophages induced by Cu and Cu-Sr modified 3D-printed scaffolds led to an increase in the inflammatory response, which was beneficial to the phagocytosis and killing of bacteria by macrophage.

In general, the increased inflammatory response activates osteoclast activity, leading to bone loss, which is detrimental to bone repair (Thomson et al., 1987; Ishimi et al., 1990). However, a moderate inflammatory environment can promote responsive proliferation and differentiation of osteoblasts. Guihard et al. (2012) reported that the IL6 family cytokine OSM released by M1 macrophages enhanced the osteogenic differentiation of bone marrow mesenchymal stem cells (BMSCs). Lu et al. (2017) also reached the same conclusion that BMSCs grown in an M1 macrophage culture medium had stronger osteogenic differentiation ability BMSCs than that in M2 macrophages. Consistent with these results, a greater ability of bone mineralization in the Cu-modified 3D printed scaffold species was obtained compared to the control group.

In addition to regulating inflammation levels, macrophages can also mediate bone regeneration by secreting osteoinductive factors (Chen et al., 2017; Jin et al., 2019). For example, bone morphogenetic protein-2 (BMP-2) and tumor inhibin M (OSM) are effective inducers of osteogenesis secreted by macrophages (Guihard et al., 2015; Wei et al., 2018). Figure 6 shows the expression levels of BMP-2 and OSM in macrophages cultured on different samples. The Cu-Sr modified 3D-printed scaffold showed higher expression levels of BMP-2 and OSM than the other scaffolds. The expression of BMP-2 was not significantly upregulated in the copper-modified 3D printing stent, while that of OSM was upregulated. Zhang et al. (2017) found that OSM was secreted by M1 macrophages, whereas higher BMP-2 secretion was derived from M2 macrophages, which was due to the existence of Sr ions interfering with the Cu ion-mediated M1 phenotype.

Therefore, the preparation of 3D printed scaffolds by double-doped copper and strontium ions enhanced the ability of bone formation and had antibacterial properties. Li et al. (2022) recently prepared titanium scaffolds with copper and strontium ions double-doped with hydroxyapatite, which inhibited bacterial growth and promoted osteogenic differentiation of BMSCs *in vitro*. Consistent with this result, Cu-Sr-modified titanium scaffolds play an antibacterial and osteogenic role by regulating macrophage polarization in our paper. The Cu and Cu-Sr modified 3D-printed scaffolds have different proinflammatory effects. Compared with the Cu-Sr modified 3D-printed scaffold, the expression level of proinflammatory cytokines in the Cu modified 3D-printed scaffold was higher, which was attributed to the strong inflammatory response caused by the massive release of copper ions. However, Sr ions in the Cu-Sr modified 3D-printed scaffold reduced the relative concentration of Cu, which led to a decrease in metal ion-mediated inflammatory reactions and the secretion of proinflammatory cytokines. From another point of view, the addition of Sr ions reduced the excessive inflammatory response and avoided apoptosis or death of immune cells as the results indicated.

Although some positive effects of Cu-Sr modified 3D printed scaffolds in immunoregulatory antibacterial and osteogenesis *in vitro* were identified, there are still some limitations in this study. First, given the complexity of the bone infection microenvironment, it is necessary to further evaluate the antimicrobial and osteogenic effects of this composite scaffold in animal bone infection models. Second, metal ions need to maintain a high concentration and long-term contact with bacteria to achieve the corresponding bactericidal effect. The doping amount of metal ions in biomaterials is closely related to their antibacterial properties. However, a high concentration of metal ions may also show unavoidable toxicity to cells and normal tissues. Therefore, the release of metal ions at a low dose to obtain a high bactericidal effect is the focus of research.

## Conclusion

Given, titanium alloy implant-related infection and implant osseointegration disorder, an immune regulation strategy was proposed to prepare Cu-Sr modified 3D-printed scaffolds that released Cu and Sr ions. Macrophage M1 polarization was induced by Cu-Sr ions to achieve immune phagocytosis of bacteria. Cu-Sr ions were employed to induce macrophages to secrete bone factors and improve osseointegration. In summary, Cu-Sr modified 3D-printed scaffolds regulated macrophage M1 polarization, osteogenic factor secretion, and other immune regulatory processes through ion release, generating excellent antibacterial and osteogenic effects. This study indicated that we could propose immunomodulatory strategies were proposed based on the immunological characteristics of target diseases, further providing ideas for the design and synthesis of new immunoregulatory biomaterials.

## Data Availability

The original contributions presented in the study are included in the article/Supplementary Material, further inquiries can be directed to the corresponding authors.
