# Supplementary material for: A surface metal ion-modified 3D-printed Ti-6Al-4V implant with direct and immunoregulatory antibacterial and osteogenic activity
Source: Front Bioeng Biotechnol. 2023 Mar 16;11:1142264. doi: 10.3389/fbioe.2023.1142264 (PMC10060813; doi:10.3389/fbioe.2023.1142264)
Supplement: Supplementary file 1 [file DataSheet1.docx]

Fig. S1. (A) The FTIR of the metal ion-modified Ti-6Al-4V implants.


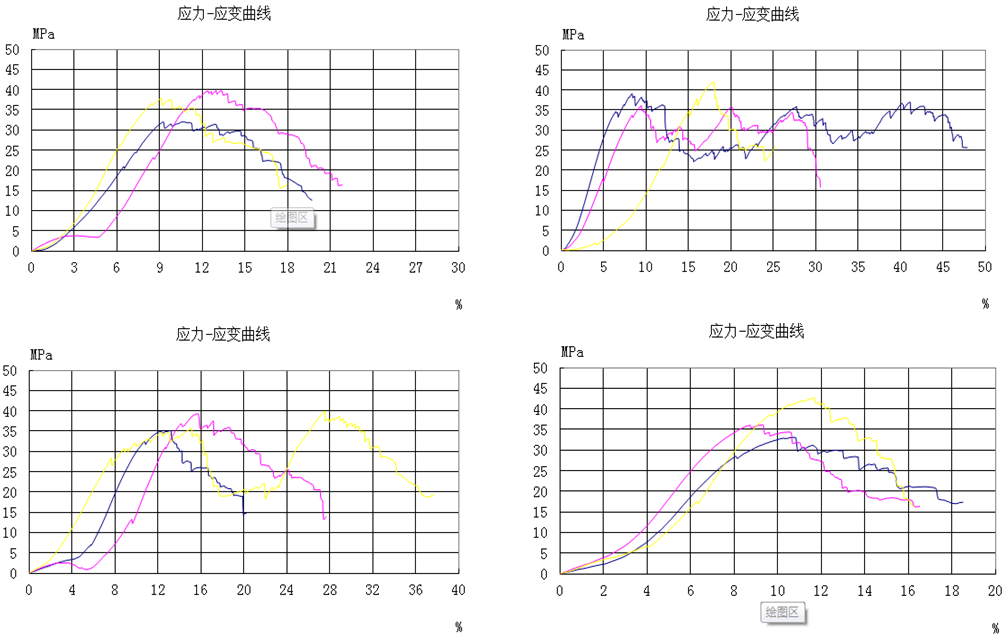


Fig. S2. Original data of stress-strain curve.


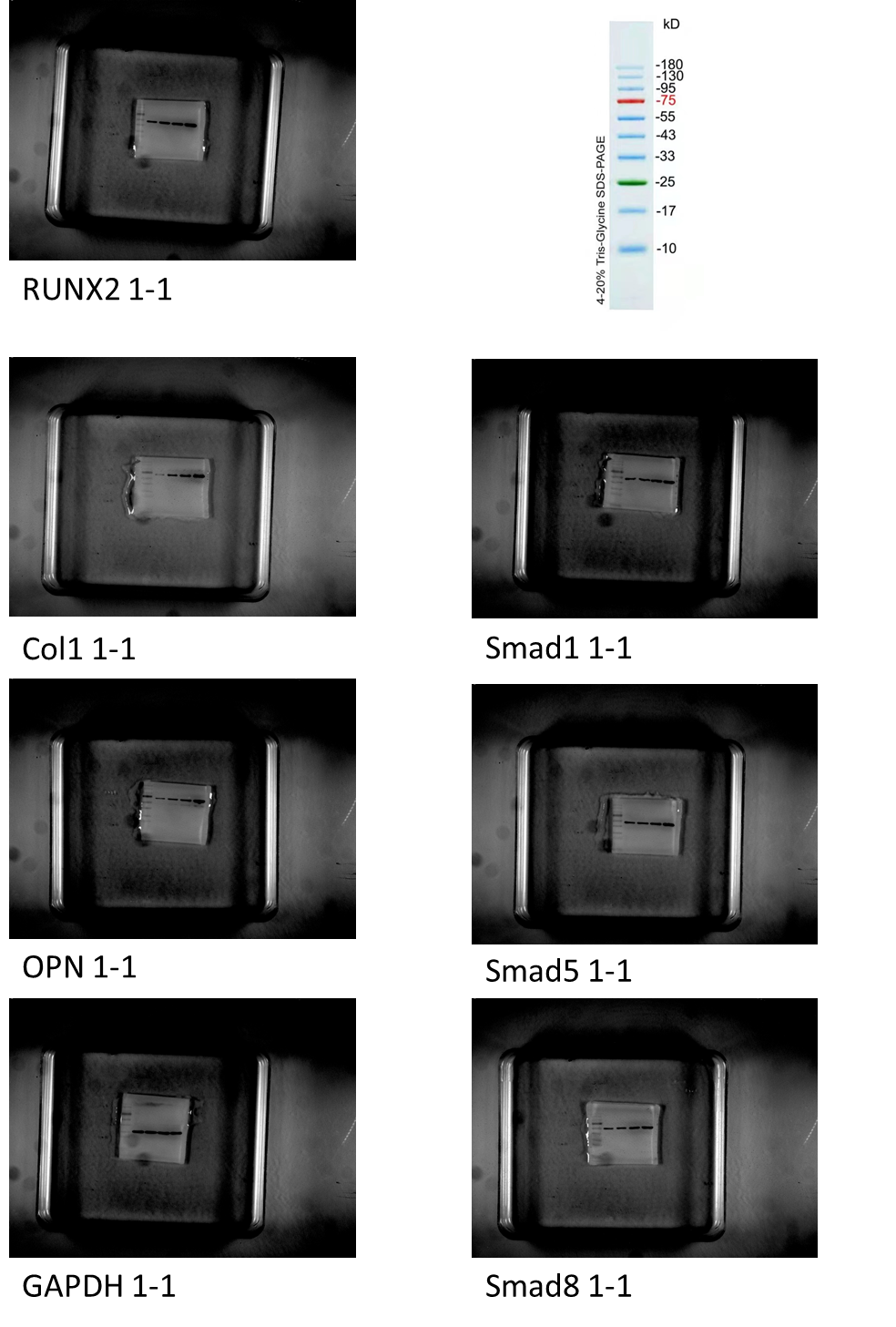


Fig. S3. Original data of immunoblotting.


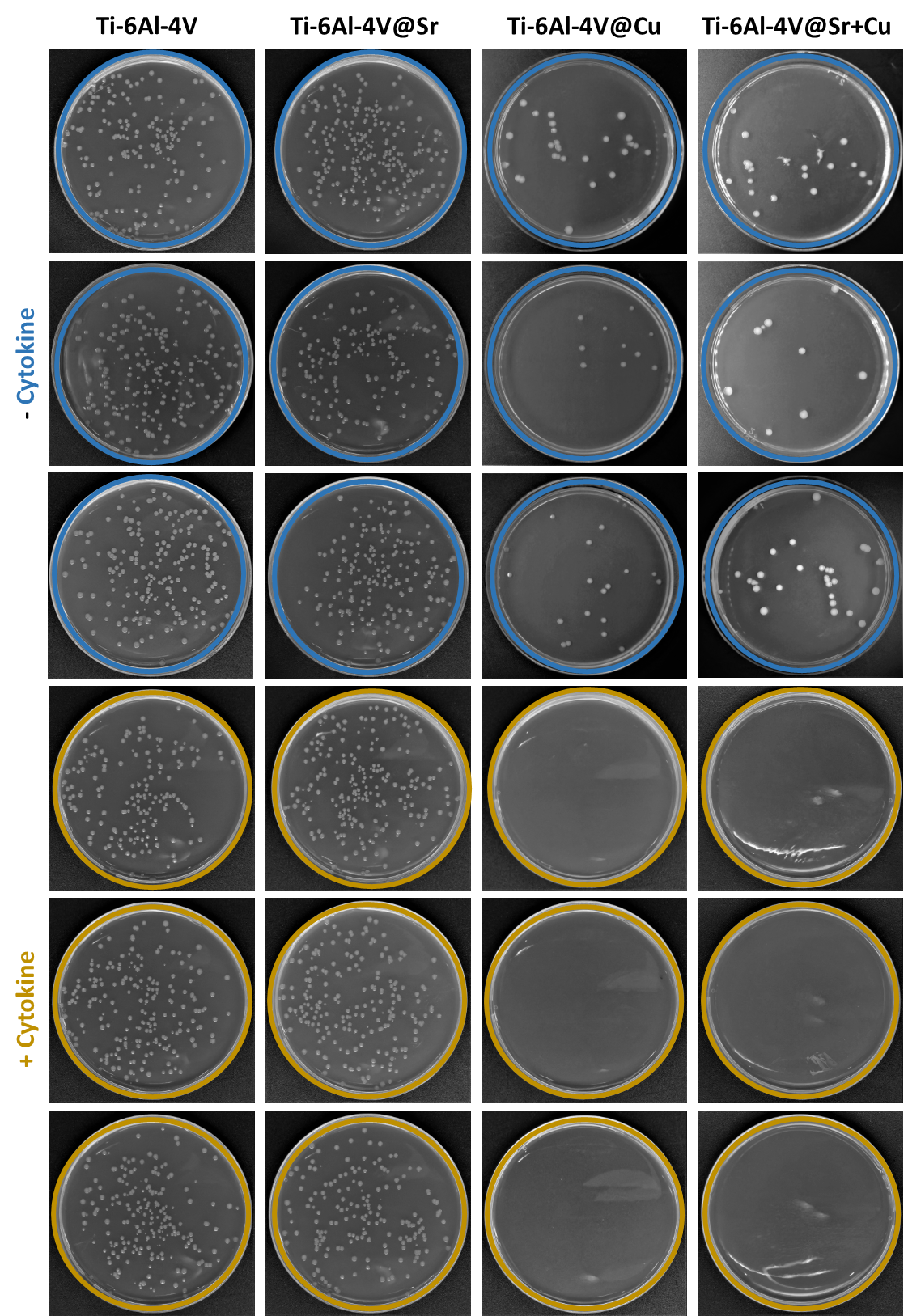


Fig. S4. Original data of bacterial coating.
